# Inactivation of Cerebral Cavernous Malformation Genes Results in Accumulation of von Willebrand Factor and Redistribution of Weibel-Palade Bodies in Endothelial Cells

**DOI:** 10.3389/fmolb.2021.622547

**Published:** 2021-07-09

**Authors:** Christiane D. Much, Barbara S. Sendtner, Konrad Schwefel, Eric Freund, Sander Bekeschus, Oliver Otto, Axel Pagenstecher, Ute Felbor, Matthias Rath, Stefanie Spiegler

**Affiliations:** ^1^ Department of Human Genetics, Interfaculty Institute of Genetics and Functional Genomics, University Medicine Greifswald, Greifswald, Germany; ^2^ Centre for Innovation Competence (ZIK) plasmatis, Leibniz Institute for Plasma Science and Technology (INP), Greifswald, Germany; ^3^ Centre for Innovation Competence (ZIK) ‐ Humoral Immune Reactions in Cardiovascular Diseases, University of Greifswald, Greifswald, Germany; ^4^ Department of Neuropathology, Center for Mind, Brain and Behavior (CMBB), University Hospital Giessen and MarburgMarburg, Germany

**Keywords:** cerebral cavernous malformation, CCM1, blood outgrowth endothelial cells, CRISPR/Cas9, von Willebrand factor

## Abstract

Cerebral cavernous malformations are slow-flow thrombi-containing vessels induced by two-step inactivation of the *CCM1*, *CCM2* or *CCM3* gene within endothelial cells. They predispose to intracerebral bleedings and focal neurological deficits. Our understanding of the cellular and molecular mechanisms that trigger endothelial dysfunction in cavernous malformations is still incomplete. To model both, hereditary and sporadic CCM disease, blood outgrowth endothelial cells (BOECs) with a heterozygous *CCM1* germline mutation and immortalized wild-type human umbilical vein endothelial cells were subjected to CRISPR/Cas9-mediated *CCM1* gene disruption. *CCM1* ^−/−^ BOECs demonstrated alterations in cell morphology, actin cytoskeleton dynamics, tube formation, and expression of the transcription factors KLF2 and KLF4. Furthermore, high VWF immunoreactivity was observed in *CCM1* ^*−/−*^ BOECs, in immortalized umbilical vein endothelial cells upon CRISPR/Cas9-induced inactivation of either CCM1, CCM2 or CCM3 as well as in CCM tissue samples of familial cases. Observer-independent high-content imaging revealed a striking reduction of perinuclear Weibel-Palade bodies in unstimulated *CCM1* ^*−/−*^ BOECs which was observed in *CCM1* ^+/−^ BOECs only after stimulation with PMA or histamine. Our results demonstrate that CRISPR/Cas9 genome editing is a powerful tool to model different aspects of CCM disease *in vitro* and that CCM1 inactivation induces high-level expression of VWF and redistribution of Weibel-Palade bodies within endothelial cells.

## Introduction

Cerebral cavernous malformations (CCMs) are convolutes of dilated capillaries in the central nervous system. Familial occurrence of multiple CCMs (OMIM 116860, 603284, 603285) is associated with autosomal-dominantly inherited loss-of-function variants in one of the three genes *CCM1* (*KRIT1,* OMIM: *604214), *CCM2* (Malcavernin, *OSM*, *607929) or *CCM3* (*PDCD10*, *TFAR15*, *609118). In agreement with a Knudsonian two-hit mechanism, somatic inactivation of the corresponding wild-type allele in vascular endothelial cells (ECs) is widely accepted as the critical step in CCM initiation (Gault et al., 2005; Akers et al., 2009; Pagenstecher et al., 2009; McDonald et al., 2014; Rath et al., 2020). In patients without a pathogenic germline variant, two biallelic somatic mutations are thought to cause CCM disease (McDonald et al., 2014).

Since the first reports of disease-causing *CCM1* variants in familial CCM cases (Laberge-le Couteulx et al., 1999; Sahoo et al., 1999), we have learned a lot about CCM pathogenesis and the endothelial dysfunction in cavernous lesions (Maddaluno et al., 2013; Cuttano et al., 2016; Zhou et al., 2016; Detter et al., 2018; Malinverno et al., 2019; Hong et al., 2020; Ren et al., 2021). A first expert consensus guideline and clinical recommendations for CCM management have been published in recent years (Akers et al., 2017; Flemming and Lanzino, 2020). However, there are still no effective drugs that would prevent cavernoma formation or CCM bleeding. A recent prospective population-based study revealed that antithrombotic therapy – anticoagulant as well as antiplatelet – is associated with a lower risk of intracranial hemorrhage and focal neurological deficits (Zuurbier et al., 2019). These counterintuitive data are consistent with a neuropathological study that anticipated a dysfunction of cavernous ECs which would result in local organizing thrombi as the primary step causing repeated secondary microhemorrhages and disease progression (Abe et al., 2005). The multimeric von Willebrand factor (VWF) protein is an important player in primary hemostasis. VWF is almost exclusively expressed by vascular ECs and bone marrow megakaryocytes (Lip and Blann, 1997). In the vascular endothelium, VWF is stored in Weibel-Palade bodies (WPBs) which secrete their prothrombotic content through exocytosis (Weibel and Palade, 1964; Sadler, 1998; Wagner and Frenette, 2008). Secretion of large amounts of VWF from WPBs can be triggered by shear stress or stimulation with Ca^2+^ raising agents like histamine, thrombin or vascular endothelial growth factor (VEGF) (Loesberg et al., 1983; Erent et al., 2007).

Using CRISPR/Cas9-based *in vitro* modeling of hereditary and sporadic CCM disease, we here demonstrate that the inactivation of CCM1, CCM2 or CCM3 in ECs induces intracellular VWF accumulation and WPB redistribution. Analyses of human CCM tissue samples support the hypothesis that increased VWF levels in the endothelium of distended caverns contribute to the local hemostatic imbalance in these fragile vascular lesions.

## Materials and Methods

### Cell Culture

The generation of blood outgrowth endothelial cells (BOECs) from a CCM proband with a pathogenic *CCM1* germline variant (*CCM1* ^*+/−*^) has been described before (Spiegler et al., 2019). Immortalized human umbilical vein endothelial cells (CI-huVECs) were obtained from InSCREENex (Braunschweig, Germany) and maintained in complete endothelial cell growth medium (ECGM; PromoCell, Heidelberg, Germany) supplemented with 10% fetal calf serum (FCS; Thermo Fisher Scientific, Waltham, MA, United States). Tube formation was performed in 384-well microplates. In brief, 17 µl Matrigel (Corning, Kaiserslautern, Germany) was incubated at 37 °C for 1 h. Next, 8,000 cells were seeded. After 16 h, tube formation was imaged and quantified with the angiogenesis analyzer for ImageJ (https://imagej.net). To stimulate secretion of WPBs, cells were grown on 96- or 6-well plates, preincubated with Opti-MEM (Thermo Fisher Scientific) for 1 h and treated solely in Opti-MEM or with dimethyl sulfoxide (DMSO, Carl Roth, Karlsruhe, Germany), 150 nM Phorbol 12-myristate 13-acetate (PMA, Cayman Chemical, Ann Arbor, MI, United States), 100 µM Histamine (Sigma-Aldrich, St. Louis, MO, United States), 1 µM Desmopressin (Acetate) (DDAVP, MedChem Express, Sollentuna, Sweden) or 1 µM DDAVP plus 100 µM 3-Isobutyl-1-methylxanthine (IBMX, Sigma-Aldrich, St. Louis, MO, United States) in Opti-MEM for 1 h according to Wang and colleagues (Wang et al., 2013).

### CRISPR/Cas9-Mediated Gene Editing

*CCM1* ^*−/−*^ BOECs were generated with CRISPR/Cas9 genome editing from *CCM1* ^*+/−*^ BOECs as described before (Spiegler et al., 2019). In brief, *CCM1* ^*+/−*^ BOECs were transfected with crRNA:tracrRNA:Cas9 ribonucleoprotein (RNP) complexes that were specific to the *CCM1* wild-type allele. Upon confluence on T25 flasks, the CRISPR/Cas9-treated cell mixture was seeded on 96-well plates with an average cell density of 0.5 cells/well. Emerging BOEC clones were genotyped by next generation sequencing in a custom two-step PCR enrichment approach. PCR products were pooled after purification with Agencourt AMPure XP beads (Beckman Coulter, Brea, CA, United States) and sequenced with 2 × 150 cycles on a MiSeq instrument (Illumina, San Diego, California, United States of America). The SeqNext software was used for data analysis (JSI Medical Systems, Ettenheim, Germany). Only variants with quality scores ≥30 were called. BOEC clones with biallelic *CCM1* loss-of-function variants (*CCM1* ^*−/−*^) were selected for further expansion. Three to four individual *CCM1* ^+/−^ BOEC lines that had been clonally expanded from the blood of the CCM proband were used as controls. For complete knockout of *CCM1*, *CCM2* and *CCM3* in CI-huVECs, the following Alt-R CRISPR-Cas9 crRNAs (Integrated DNA Technologies (IDT), Coralville, Iowa, United States of America) were used: 5′-GGA​GCT​CCT​AGA​CCA​AAG​TA-3′ (*CCM1*), 5′-GGT​CAG​TTA​ACG​TCC​ATA​CC-3' (*CCM2*), and 5′-CAA​CTC​ACC​TCA​TTA​AAC​AC-3' (*CCM3*). A non-targeting crRNA (nc crRNA #1, IDT) served as control. Reverse transfection, estimation of the genome editing efficiencies by T7 endonuclease I digestion or Sanger sequencing, and the expansion of knockout CI-huVEC clones were performed as delineated previously (Schwefel et al., 2019; Spiegler et al., 2019; Schwefel et al., 2020).

### Immunofluorescence Analyses

After fixation of the cells on 96-well plates with 4% paraformaldehyde for 10 min, permeabilization, and several washing steps, immunofluorescent analyses were performed using polyclonal rabbit anti-human KLF4 (1:100, PA5-27441, Thermo Fisher Scientific), rabbit anti-vascular endothelial (VE)-cadherin (1:100, ab33168, Abcam, Cambridge, United Kingdom), CytoPainter Phalloidin-iFluor 488 (1:1,000, ab176753, Abcam), mouse anti-human KLF2 (1:88, MAB5466, R&D Systems, Minneapolis, Minnesota, United States of America), monoclonal mouse anti-human vWF (1:100, MA5-14029, Thermo Fisher Scientific), Alexa Fluor 488-conjugated secondary goat anti-mouse IgG antibody (A-11029, Thermo Fisher Scientific), Alexa Fluor 555-conjugated goat anti-mouse IgG antibody (ab150114, Abcam) or Alexa Fluor 555-conjugated goat anti-rabbit IgG antibody (A-21429, Thermo Fisher Scientific). DAPI (D9542, Sigma-Aldrich) was used to stain cell nuclei and image acquisition was performed with either an EVOS FL (Thermo Fisher Scientific) or a Zeiss LSM980 Airyscan 2 microscope (Zeiss, Jena, Germany) after addition of mounting medium (50001, Ibidi, Gräfelfing, Germany). After the sensitivity and specificity of VWF staining had been verified, the focus was placed on the *CCM1* ^−/−^ cells in direct comparisons to avoid overexposure.

### High-Content Imaging

Quantification of WPBs was performed on cells grown on glass bottom imaging plates (Eppendorf, Hamburg, Germany) and following secretagogues treatment using a high-content imaging microscope (Operetta CLS; Perkin Elmer, Waltham, MA, United States). In addition to the digital phase contrast channel (pseudo-cytosolic signal), the images were acquired in three z-planes to detect VWF-positive granula (λex 475 nm/λem 500–550 nm) and DAPI-stained nuclei (λex 365 nm/λem 430–500 nm). The algorithm-driven image quantification was performed utilizing the Harmony 4.9 (Perkin Elmer) imaging and analysis software. The images were combined with their maximum projection intensities from all three z-planes and flatfield-adjusted. Per treatment and cell type, at least 2,500 cells in a total of 27 fields of view have been included in the analysis. The nuclei were segmented by their DAPI signal, followed by detection of the surrounding cytosolic area. A selection step was applied to exclude border region objects and dead cells with fragmented nuclei. In order to enhance the contrast, a sliding parabola function was applied, which allowed better discrimination of bright objects. Afterward, the number of WPBs and their intensity was determined inside the cytosolic cell region. To assess the amount of WPBs near the cell nucleus, a 5 µm ring-region was calculated and the granules were quantified inside this region.

### Biochemical Analyses

To quantify the secretion of VWF, cell culture supernatants were collected and concentrated (Vacuum concentrator centrifuge, UniEquip, Planegg, Germany). Proteins were analyzed in duplicates using Human VWF-A2 DuoSet ELISA (DY2764-05, R&D Systems) and Ancillary Reagent Kit (DY008, R&D Systems) according to the manufacturer’s protocol. Absorbance at 450 and 540 nm was measured on a Paradigm platform (Beckman Coulter, Brea, CA, United States). Values were corrected for 540 nm and normalized to untreated cells.

### Immunohistochemistry on Tissue Samples

Immunohistochemistry was performed on FFPE tissue slices as described before (Pagenstecher et al., 2009) using vWF antibody (1:100, M616, Dako (Agilent)) following heat induced antigen retrieval in citrate buffer. Staining intensity in the endothelia of CCMs and vessels in the surrounding brain was evaluated semiquantitatively using five tiers ranging from 0 to ++++.

### Statistical Analyses

Data analysis was performed using GraphPad prism software (GraphPad Software, San Diego, CA, United States). Multiple t tests or two-way ANOVA were used for statistical analysis. Where appropriate, Šidák or Dunnet corrections for multiple comparisons were used. For quantification of ELISA data, VWF concentrations were calculated from the standard curve by linear regression performed with the Origin software (Northampton, MA, United States). The standard curve was obtained by fitting the Hill equation [y=Vmax*xn/(kn+xn)] to our data, with *Vmax* = 6.00716, *k* = 8,210.36, *n* = 0.72, and *R2* = 0.99319. *p < 0.05, **p < 0.01, ***p < 0.001, ****p < 0.0001.

## Results

### Modeling Hereditary and Sporadic CCM Disease *In Vitro*

**FIGURE 1:**
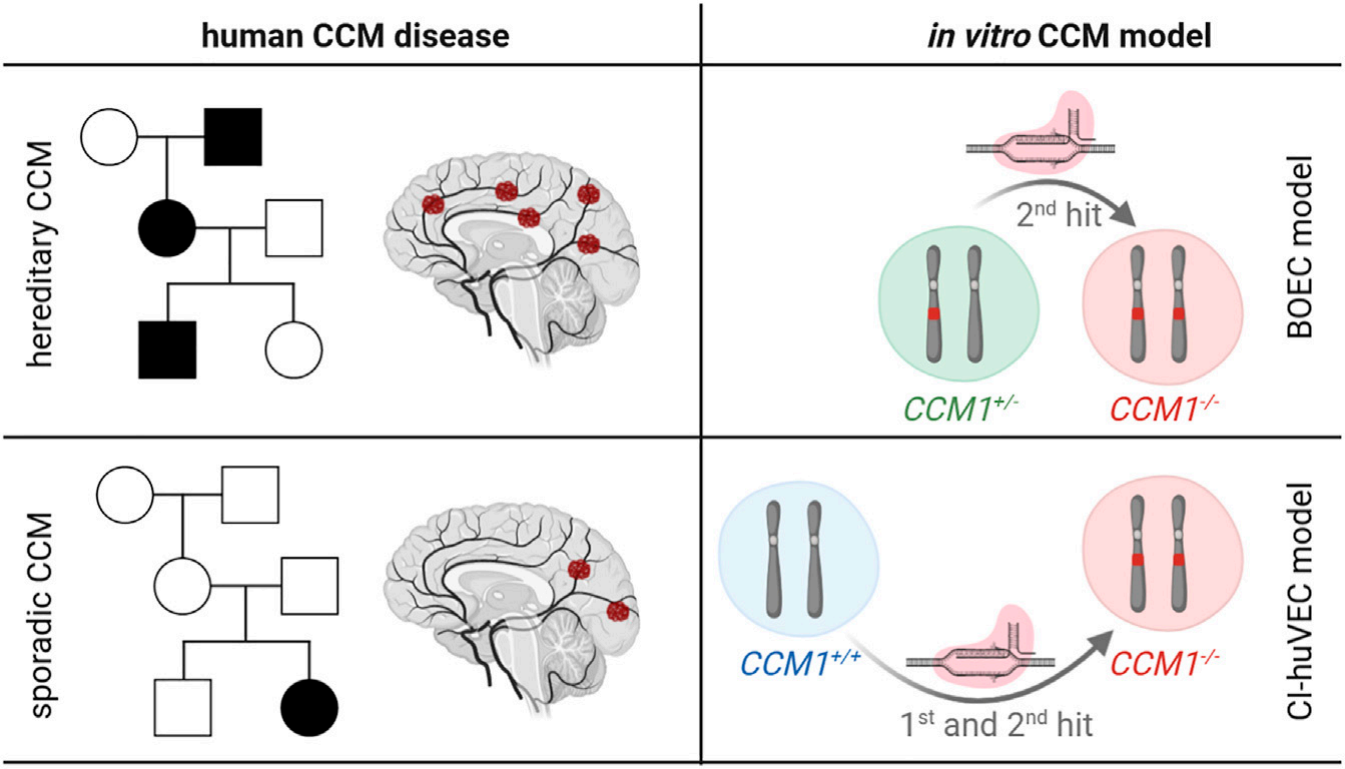
Overview of CCM cell culture models. Features of hereditary and sporadic CCM disease (left subpanels) as well as the CRISPR/Cas9 genome editing approaches that have been used in this study (right subpanels) are schematically depicted (created with BioRender.com). Filled symbols in the pedigrees indicate CCM patients. *CCM1* ^*+/+*^ *=* cells with two *CCM1* wild-type alleles*; CCM1* ^+/−^ = cells with a heterozygous *CCM1* mutation; *CCM1* ^−/−^ = cells with biallelic *CCM1* mutations.

To model the hereditary and sporadic type of CCM disease *in vitro* (Figure 1), we used BOECs isolated from peripheral blood of a CCM proband with a heterozygous *CCM1* loss-of-function germline variant (*CCM1* ^*+/−*^) and wild-type CI-huVECs (*CCM1* ^+/+^)*.* Using CRISPR/Cas9 genome editing, we inactivated the *CCM1* wild-type allele in *CCM1* ^*+/−*^ BOECs or induced biallelic frameshift variants in *CCM1* ^+/+^ CI-huVECs and thereby generated pairs of *CCM1* ^*+/−*^ and *CCM1* ^*−/−*^ BOECs (hereditary CCM model) or *CCM1* ^*+/+*^ and *CCM1* ^*−/−*^ CI-huVECs (sporadic CCM model), respectively.

### *CCM1* ^*−/−*^ BOECs Reflect Key Features of CCM Disease

**FIGURE 2:**
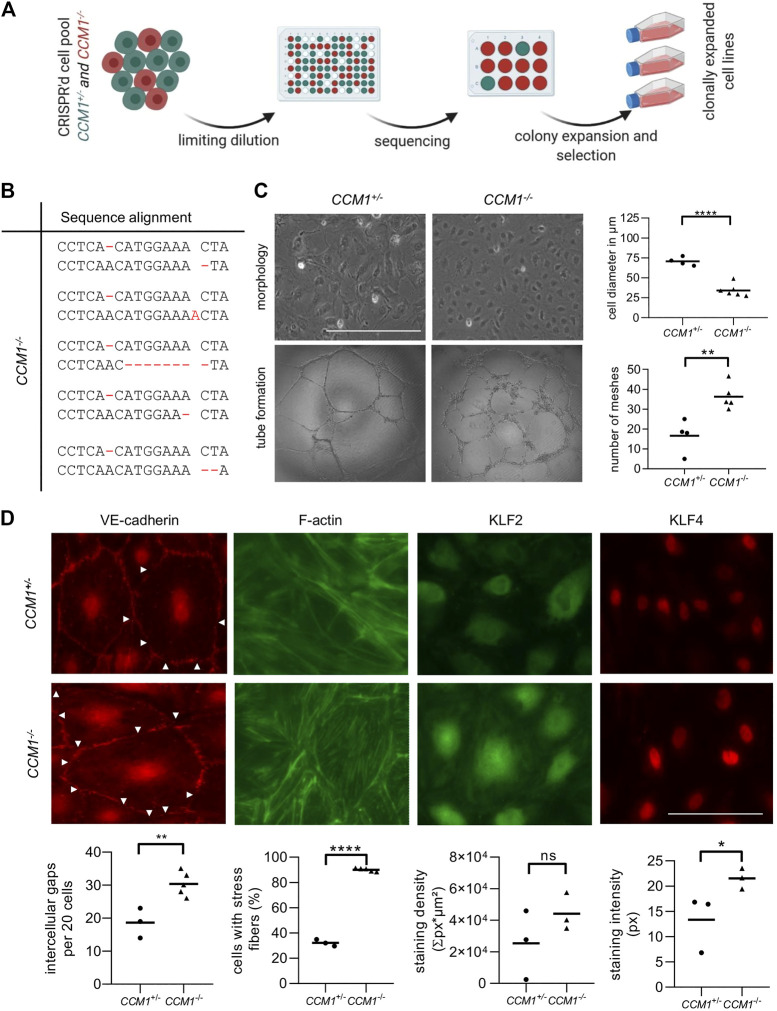
Characterization of *CCM1* ^+/−^ and clonally expanded *CCM1* ^−/−^ BOECS. **(A)** *CCM1* ^−/−^ BOECs were established by limiting dilution of the CRISPR/Cas9 RNP-treated cell pool and clonal expansion of single cells (created with BioRender.com). **(B)** Shown are the genotypes of *CCM1* ^*−/−*^ BOEC clones included in this study. All variants either lead to a frameshift or a premature stop codon (additional information on the CRISPR/Cas9-induced mutations and numbers of individual clones are given in Supplementary Table S1). **(C)** *CCM1* ^−/−^ BOECs presented a more compact morphology in brightfield microscopy and increased tube formation. The largest cell diameter and the number of meshes formed on Matrigel-coated plates were quantified. Scale bar indicates 400 µm. Data are presented as single data points with the mean (*n* = 4–6). **(D)** Immunofluorescence staining revealed a higher number of intercellular gaps (white arrowheads), more actin stress fibers, and a higher expression of KLF2 and KLF4 in *CCM1* ^−/−^ BOECs. Representative images are shown. Scale bar indicates 100 µm. Individual data points are shown with the mean (*n* = 3–5). Student’s *t* test was used for statistical analysis: *p < 0.05; **p < 0.01; ****p < 0.0001; ns = not significant; VE-cadherin = vascular endothelial cadherin.

To study the effects of second-hit inactivation of *CCM1* in human ECs, we first established thirty clonal *CCM1* ^*−/−*^ BOEC lines in a single cell cloning approach (Figure 2A). NGS amplicon sequencing verified compound heterozygosity for the pre-existing *CCM1* germline variant and a second CRISPR/Cas9-induced mutation that led to inactivation of the *CCM1* wild-type allele (Figure 2B, Supplementary Table S1). Further cell line characterizations demonstrated striking differences between *CCM1* ^−/−^ and *CCM1* ^*+/−*^ BOECs in terms of cell morphology, angiogenic properties, integrity of intercellular junctions, and organization of the actin cytoskeleton. While *CCM1* ^+/−^ BOECs had a cell diameter of 70 µm in two-dimensional monolayer culture, *CCM1* ^−/−^ BOECs presented a more compact morphology with a diameter of only 34.2 µm. In addition, the number of meshes formed by *CCM1* ^−/−^ BOECs on Matrigel-coated plates was significantly increased by 118% (Figure 2C). We also examined the integrity of endothelial adherens junctions as this is a main determinant of vascular permeability (Figure 2D). Remarkably, VE-cadherin staining revealed a 62,6% increase of intercellular gaps upon second-hit inactivation of *CCM1* in BOECs. While stress fibers (SF) were found in only 32% of *CCM1* ^+/−^ BOECs, over 90% of CCM1-deficient BOECs were SF-positive (Figure 2D). The misregulation of actin cytoskeleton dynamics in *CCM1* ^−/−^ BOECs further reinforced that our BOEC model reflects key features of CCM pathophysiology. Since the zinc finger transcription factors KLF2 und KLF4 have been reported as drivers of CCM disease (Renz et al., 2015; Zhou et al., 2016), we decided to address KLF2/4 expression in our *in vitro* model of hereditary CCM disease. KLF4 levels were significantly increased in *CCM1* ^−/−^ BOECs (Figure 2D). Furthermore, a significant upregulation of *KLF2* mRNA (Supplementary Figure S1), and a trend towards increased KLF2 expression on protein level were found in *CCM1* ^−/−^ BOECs (Figure 2D).

### *CCM1* Gene Disruption Induces High-Level VWF Expression and WPB Redistribution in BOECs

**FIGURE 3:**
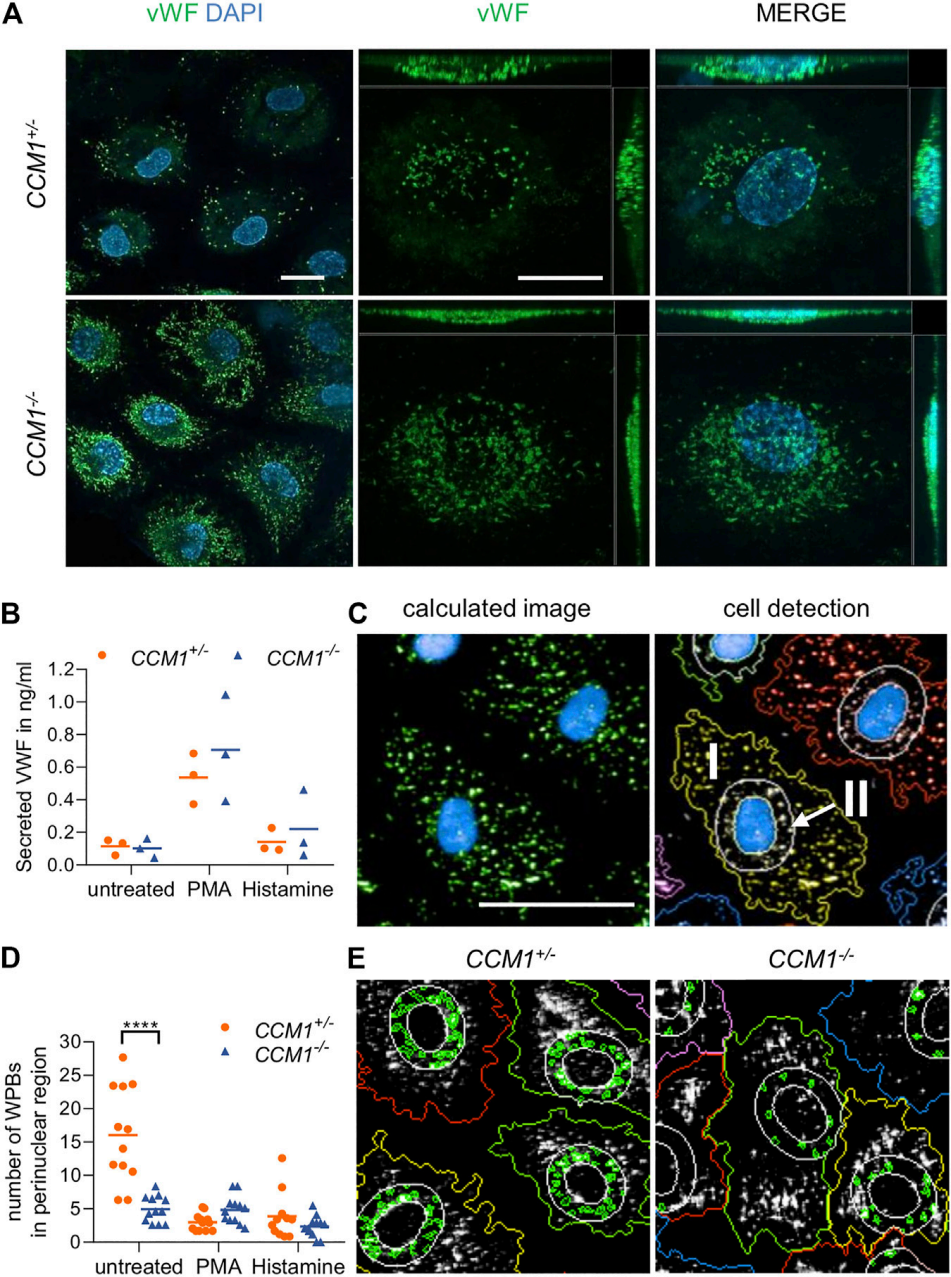
High VWF content and aberrant WPB distribution in *CCM1* ^*−/−*^ BOECs. **(A)** Immunofluorescent staining demonstrated high-level VWF expression (green) in clonally expanded *CCM1* ^*−/−*^ BOECs. DAPI (blue) was used as nuclear counterstain. Confocal images were acquired using a 63x (NA 1.4) oil objective (left) and at higher magnification shown as maximum intensity projection of image stacks (0.2 µm z planes). Scale bars indicate 20 µm. **(B)** Absolute amount of secreted VWF from *CCM1* ^*+/−*^ and *CCM1* ^*−/−*^ BOECs as quantified by ELISA. Data are presented as single data points with the mean (*n* = 3). **(C)** Analysis strategy of high-content imaging for untreated and stimulated BOECs. As basis for quantification, a sliding parabola function was applied for contrast enhancement of the VWF signal (green). Cell nuclei were segmented by their DAPI signal (blue) and the surrounding cytosolic area and perinuclear region were detected. VWF-positive granules in the cytosol (I) and the perinuclear region (II) were quantified. Scale bar indicates 30 µm. **(D)** WPBs in the perinuclear region were quantified as shown in untreated *CCM1* ^+/−^ and *CCM1* ^−/−^ BOECs. Data are presented as single data points with the mean. Two-way ANOVA with Šidák correction for multiple comparisons was used for statistical analysis. *p* < 0.05. All experiments were performed in triplicates and four biological replicates. **(E)** Representative images of WPBs in the perinuclear region in untreated *CCM1* ^+/−^ **(left)** and *CCM1* ^−/−^ BOECs **(right)**.

Immunofluorescence staining unexpectedly revealed a significant enrichment of the endothelial marker protein VWF in clonally expanded *CCM1* ^−/−^ BOECs when compared to *CCM1* ^+/−^ BOECs (Figure 3A). Targeted next generation sequencing of all coding exons and exon-intron junctions revealed no *VWF* variant which might affect gene expression or VWF secretion. Since the endothelium is the main source of plasma VWF (Nichols et al., 2008), we asked the question of whether VWF secretion might be impaired upon complete CCM1 inactivation in human ECs. Hence, VWF levels in cell culture supernatants were analyzed by ELISA. Notably, the responsiveness of *CCM1* ^*−/−*^ BOECs to stimulation with histamine which triggers Ca^2+^-mediated VWF secretion and the potent non-physiological secretagogue PMA was intact and the secreted VWF levels were not significantly different between *CCM1* ^*+/−*^ and *CCM1* ^*−/−*^ BOECs (Figure 3B). To determine alterations of the morphology and intracellular distribution of WPBs upon second-hit inactivation of *CCM1*, their length and the number of WPBs in the perinuclear region were quantified by observer-independent high-content imaging (Figure 3C). WPBs were slightly longer in *CCM1* ^*−/−*^ BOECs (2.84 µm vs. 2.76 µm under basal conditions, *p* = 0.001; data not shown). Interestingly, a perinuclear accumulation of WPBs was found in unstimulated *CCM1* ^*+/−*^ BOECs. Stimulation with histamine, PMA or the vasopressin analog DDAVP which triggers cAMP-mediated exocytosis, reduced the number of WPBs in the perinuclear region of *CCM1* ^*+/−*^ BOECs (Figures 3D,E and Supplementary Figure S2). In unstimulated *CCM1* ^*−/−*^ BOECs, however, WPBs did not accumulate in the perinuclear region (Figures 3D,E). Additionally, only histamine treatment, but not stimulation with either PMA or DDAVP, induced an additional translocation of WPBs and further reduced their number in the perinuclear region of *CCM1* ^*−/−*^ BOECs (Figures 3D,E and Supplementary Figure S2).

In conclusion, the CRISPR/Cas9-induced second-hit inactivation of *CCM1* had caused a VWF reduction in the perinuclear region comparable to the level seen after stimulation with secretagogues in heterozygous *CCM1* ^*+/−*^ BOECs. These changes most likely reflect a constitutively activated state of *CCM1* ^*−/−*^ BOECs.

### High VWF Levels in CI-huVECs after CCM1/2/3 Protein Inactivation and in the Endothelium of Human CCMs

**FIGURE 4:**
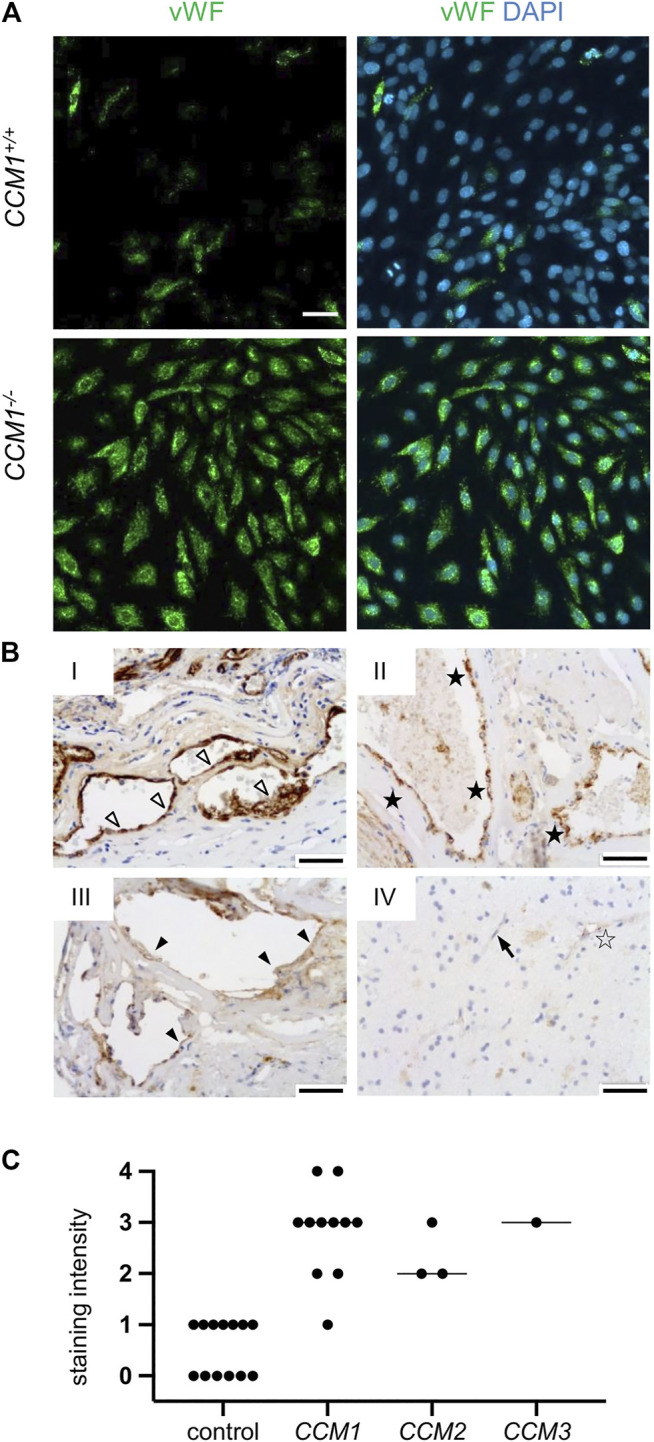
High-level VWF expression is a common feature of CCM disease. **(A)** Strong immunopositivity for VWF (shown in green) was also found in clonally expanded *CCM1* ^*−/−*^ CI-huVECs. DAPI (blue) was used as nuclear counterstain. Confocal images were acquired using a 10x (NA 0.45) objective. Scale bar indicates 50 µm. **(B)** Immunohistochemistry demonstrated medium to strong VWF staining intensities (SI) in CCM tissue samples of hereditary cases (I-III). IV = normal brain. Representative images are shown. Open arrowhead = [SI] 4, black asterisk = [SI] 3, filled arrowhead = [SI] 2, open asterisk = [SI] 1, arrow = [SI] 0. Scale bars indicate 50 µm. The graph displays the staining intensity of normal brain vessels in the vicinity of CCMs (*n* = 13) and of cavernous vessel endothelia in *CCM1* (*n* = 11), *CCM2* (*n* = 3), and *CCM3* (*n* = 1) probands. Bars indicate the median.

VWF levels have recently been reported as marker for the proliferation capacity of individual BOEC clones (de Boer et al., 2020) which are also called endothelial colony forming cells (Nowak-Sliwinska et al., 2018). Furthermore, changes in VWF expression might also indicate different stages of aging and endothelial-to-mesenchymal transition of BOECs in cell culture (de Jong et al., 2019; de Boer et al., 2020). In a next step, we therefore used immortalized CI-huVECs which represent a well characterized EC culture model (Heiss et al., 2015; Lipps et al., 2018). In line with our observations in *CCM1* ^*−/−*^ BOECs, clonally expanded *CCM1* ^*−/−*^ CI-huVECs presented strong immunopositivity for VWF (Figure 4A). The same outcome was seen in CI-huVECs that had been treated with *CCM1*-, *CCM2*- or *CCM3*-specific crRNA:tracrRNA:Cas9 RNPs (Supplementary figure S3).

Finally, we validated our *in vitro* data in formalin-fixed, paraffin-embedded CCM tissue samples of fifteen probands with a pathogenic germline variant in either *CCM1*, *CCM2* or *CCM3* and loss of CCM protein expression in the CCM endothelium (Pagenstecher et al., 2009). In accordance with our *in vitro* data, immunohistochemical analyses demonstrated high VWF signals in endothelial cells lining distended caverns (Figures 4B,C).

## Discussion

Identifying a molecular explanation for the bleeding tendency of CCMs has been defined as one of the top research priorities by patients and health-care providers (Al-Shahi Salman et al., 2016). Following this recommendation, we here demonstrate the value of CRISPR/Cas9 genome editing in modeling hereditary and sporadic CCM disease *in vitro*, add VWF to the growing list of molecules involved in CCM pathogenesis, and support the hypothesis that a local hemostatic imbalance contributes to thrombosis and hemorrhage in CCMs.

Genome editing has become a powerful tool to model complex human diseases *in vitro*. We have previously used the CRISPR/Cas9 technology for targeted correction of the *CCM1* mutant allele and second-hit inactivation of *CCM1* in *CCM1* ^+/−^ BOECs. However, the low clonogenicity of *CCM1* ^+/+^ BOECs and the survival advantage of *CCM1* ^−/−^ BOECs hampered our efforts to fully mimic CCM lesion genesis *in vitro* (Spiegler et al., 2019). Hence, we have now combined our BOEC model which perfectly reproduces the two-hit inactivation mechanism of hereditary CCM (*CCM1* ^+/−^ vs. *CCM1* ^−/−^) with our CI-huVEC model which mimics sporadic CCM disease (*CCM1* ^+/+^ vs. *CCM1* ^−/−^). Well-known features of CCM pathobiology such as actin stress fiber formation, disruption of the integrity of intercellular junctions or upregulation of the transcription factors KLF2 and KLF4 (Glading et al., 2007; Schneider et al., 2011; Faurobert et al., 2013; Shenkar et al., 2015; Cuttano et al., 2016; Zhou et al., 2016) were recapitulated in this cell culture model.

Furthermore, we addressed a new aspect of the hemostatic imbalance in CCMs. CCM bleeding is one of the major concerns of CCM patients and their doctors. Since the risk of future bleeding events is even increased after a first hemorrhage (Al-Shahi Salman et al., 2012), the existence of an anticoagulant micromilieu in CCMs seems plausible (Lopez-Ramirez et al., 2019). However, the frequent observation of thrombi in caverns and nonhemorrhagic focal neurological deficits in CCM patients (Abe et al., 2005; Al-Shahi Salman et al., 2008; Cortes Vela et al., 2012; Horne et al., 2016) suggest a more complex interplay of pro- and anticoagulatory processes in CCM disease. High VWF levels in ECs upon *CCM1*, *CCM2* or *CCM3* gene disruption and, most importantly, the striking VWF immunopositivity within the lining endothelium of individual caverns of human CCMs support this hypothesis. Since stimulation with secretagogues induced proper VWF secretion in *CCM1* ^*−/−*^ BOECs, it is reasonable to assume that vascular stasis and transient procoagulant stimuli trigger local thrombus formation in cavernous lesions. The high affinity of thrombin for the anticoagulant endothelial receptor thrombomodulin which is strongly expressed on ECs of capillaries in the wall of cavernous blood vessels (Abe et al., 2005), may, on the other hand, confer limited protection against thrombosis at the cost of potential bleeding (Lopez-Ramirez et al., 2019). Loose intercellular junctions and recapillarization of organizing thrombi may further promote repeated microhemorrhages into the neighboring brain tissue.

VWF secretion and WPB distribution inside ECs are controlled by a highly regulated molecular network. The secretagogues histamine, DDAVP, and PMA used in this study cover the most important signaling cascades of WPB exocytosis from human ECs (Schillemans et al., 2019). Remarkably, stimulation of *CCM1* ^*−/−*^ BOECs with these secretagogues demonstrated that the second-hit inactivation of *CCM1* does not attenuate the regulated exocytosis of WPBs. Although we cannot exclude that other stimuli such as forskolin, epinephrine, thrombin or VEGF (Rondaij et al., 2006) might have a different effect on CCM1-deficient ECs, our observations point to increased intracellular storage of VWF upon CCM1 inactivation. Besides cAMP- or Ca^2+^-dependent pathways, Rab proteins that are recruited to WPB, the dynein-dynactin complex, the SNARE machinery, the transcription factor KLF2, and various other signaling pathways influence the formation and secretion of WPBs (Rondaij et al., 2006; van Agtmaal et al., 2012; Nightingale and Cutler, 2013; Mourik and Eikenboom, 2017). The observations from our present study suggest that CCM1 modulates this dynamic process in a KLF2-dependent manner. Upregulation of *KLF2* is a key feature of CCM disease and was also observed in our *in vitro* model. Its misexpression interferes with endothelial quiescence and increases Rho as well as ADAMTS activity (Renz et al., 2015; Zhou et al., 2016). Lentiviral overexpression of KLF2 in BOECs has been shown to increase the number of WPBs and reduce perinuclear WPB clustering (van Agtmaal et al., 2012). Furthermore, an increase in *VWF* mRNA with a simultaneous decrease in *ANGPT2* mRNA was described in HUVECs upon KLF2 overexpression (Dekker et al., 2006). These are observations that were also made in our *CCM1* ^*−/−*^ BOEC model. Despite upregulation of KLF2 in *CCM1* ^*−/−*^ BOEC, we did not observe a shortening of WPBs which would have been indicative of reduced activity of primary hemostasis (Ferraro et al., 2016; Ferraro et al., 2020). In fact, we even found slightly longer WPBs in CCM1-depleted ECs. An increased fraction of longer WPBs can be associated with a higher prothrombotic potential because they contain substantial VWF amounts (Ferraro et al., 2014; Ferraro et al., 2016). It is probably selective exocytosis of these long WPB that might be affected by CCM1 depletion. The group of Daniel Cutler recently demonstrated that the release of large WPBs requires the recruitment of an actin ring while the exocytosis of smaller WPBs does not (McCormack et al., 2020). Interestingly, reorganization of the actin cytoskeleton, particularly the decrease of cortical actin filaments and the increased formation of actin stress fibers, is one of the most studied effects of CCM protein depletion in human ECs (Figure 2D); (Glading et al., 2007; Faurobert et al., 2013; Schwefel et al., 2019; Spiegler et al., 2019; Schwefel et al., 2020). Specific actin-dependent exocytosis alterations might also be an explanation why the secretion of other WPB components can be normal or even increased in CCM1-, CCM2- or CCM3-depleted ECs as these smaller molecules can also be released by kiss-and-run or actin-independent full fusion.

In conclusion, our results point to the co-existence of pro- and anticoagulatory processes in CCM which provides an explanation for the favorable outcome seen in CCM patients who got antithrombotic therapies for other reasons (Schneble et al., 2012; Zuurbier et al., 2019). Future randomized trials will have to show whether therapies addressing hemostasiological homeostasis are not only safe for CCM patients but also have a therapeutic effect on the bleeding tendency of their CCMs.

## Data Availability

The original contributions presented in the study are included in the article/Supplementary Material, further inquiries can be directed to the corresponding author.
