# Supplementary material for: Inactivation of Cerebral Cavernous Malformation Genes Results in Accumulation of von Willebrand Factor and Redistribution of Weibel-Palade Bodies in Endothelial Cells
Source: Front Mol Biosci. 2021 Jul 9;8:622547. doi: 10.3389/fmolb.2021.622547 (PMC8298835; doi:10.3389/fmolb.2021.622547)
Supplement: Supplementary file 1 [file DataSheet1.PDF]

Supplementary Material

1 Supplementary Material and Methods

RNA analysis

200 ng total RNA was reverse transcribed with the SuperScript IV First-Strand Synthesis System (Thermo Fisher). *VWF*, *KLF2*, and *ANGPT2* gene expression differences were analyzed by quantitative PCR using Maxima SYBR Green qPCR Master Mix (Thermo Fisher) on a LightCycler 480 instrument (Roche, Mannheim, Germany) with transcript-specific primers. *RPLP0* was used as housekeeping gene.

2 Supplementary Figures

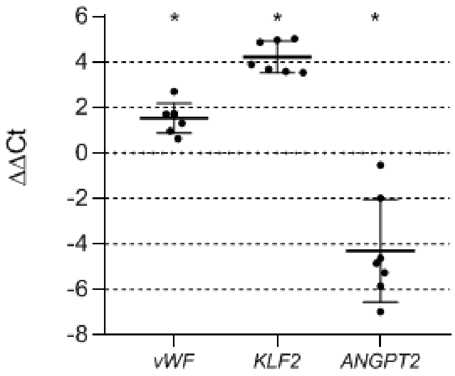

Figure S1: *VWF*, *KLF2*, and *ANGPT2* gene expression changes in *CCM1*$^{-/-}$ BOECs. qPCR analyses demonstrated upregulation of *VWF* and *KLF2* and downregulation of *ANGPT2* mRNA in *CCM1*$^{-/-}$ BOECs. Shown are ΔΔCt values. Four *CCM1*$^{+/-}$ BOEC clones served as controls. * = $p < 0.05$.

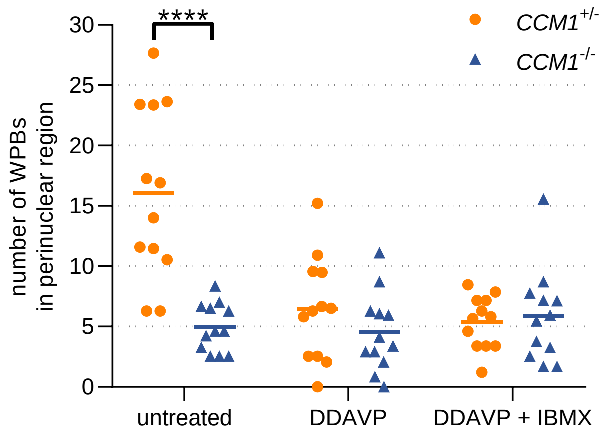

Figure S2: WPB redistribution in BOECs after stimulation with DDAVP and IBMX. Untreated *CCM1*$^{+/-}$ BOECs demonstrated perinuclear accumulation of WBPs. After stimulation with 1 µM DDAVP alone or supplemented with 100 µM IBMX, a reduced number of WBPs was found in the perinuclear region. No accumulation of WPBs was seen in *CCM1*$^{-/-}$ BOECs. Data are presented as single data points with the mean. Two-way ANOVA with Šidák correction for multiple comparisons was used for statistical analysis. $p < 0.05$. All experiments were performed in triplicates and four biological replicates. Data for DDAVP, DDAVP+IBMX, PMA and histamine stimulation were collected in the same experiment. Therefore, data for untreated BOECs are the same as shown in Figure 3D.

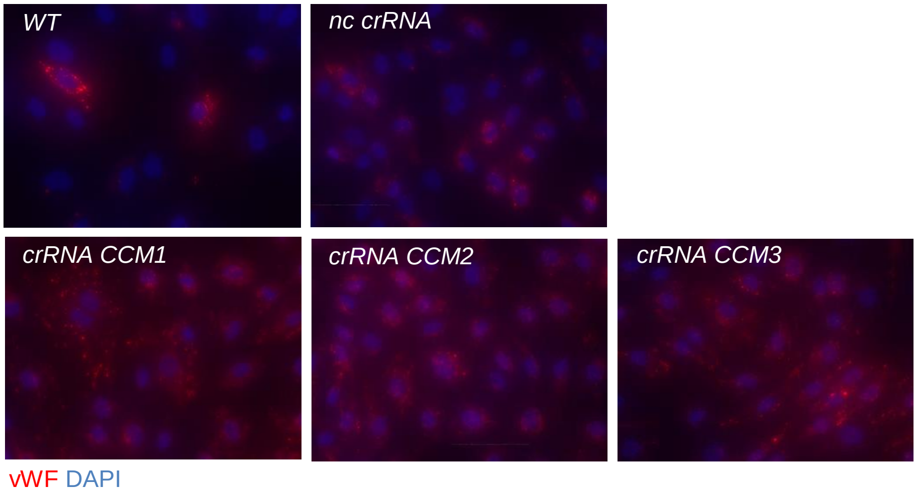

Figure S3: High-level VWF expression in CI-huVECs upon *CCM1*, *CCM2* and *CCM3* inactivation. Immunofluorescent staining demonstrated high VWF levels (red) in CI-huVECs that had been treated with *CCM1*-, *CCM2*- and *CCM3*-specific crRNA:tracrRNA:Cas9 RNP complexes. DAPI (blue) was used as nuclear counterstain. Images were acquired at 40x magnification. The scale bar indicates 50 µm. Notably, the focus was set on crRNA *CCM1*, crRNA *CCM2* and crRNA *CCM3* CI-huVECs to avoid overexposure. This results in only seemingly low VWF signals in wild-type (WT) CI-huVECs and in CI-huVECs that had been treated with a non-targeting control crRNA (nc crRNA).

3 Supplementary Tables

Table S1: Genotypes of the BOEC clones used in this study.

Genotype	Sequence	Nucleotide and protein change	Number of clones
CCM1$^{+/-}$	CCTCAACATGGAAA CTA<br>CCTCA-CATGGAAA CTA	c.[2012=];[2012del]<br>p.[Asn671=];[Asn671Thrfs*36]	4
CCM1$^{-/-}$	CCTCA-CATGGAAA CTA<br>CCTCAACATGGAAA -TA	c.[2012del];[2021del]<br>p.[Asn671Thrfs*36];[Thr674Ilefs*33]	3
	CCTCA-CATGGAAA CTA<br>CCTCAACATGGAAAACTA	c.[2012del];[2020dup]<br>p.[Asn671Thrfs*36];[Thr674Asnfs*2]	18
	CCTCA-CATGGAAA CTA<br>CCTCAAC------- -TA	c.[2012del];[2014_2021del]<br>p.[Asn671Thrfs*36];[Met672*]	2
	CCTCA-CATGGAAA CTA<br>CCTCAACATGGAA- CTA	c.[2012del];[2020del]<br>p.[Asn671Thrfs*36];[Thr674Leufs*33]	6
	CCTCA-CATGGAAA CTA<br>CCTCAACATGGAAA --A	c.[2012del];[2021_2022del]<br>p.[Asn671Thrfs*36];[Thr674Lysfs*8]	1
